# Perception and Impact of Food and Beverage Marketing on Children’s Eating Behaviors and Associated Health Issues

**DOI:** 10.7759/cureus.61210

**Published:** 2024-05-28

**Authors:** Anshoo Agarwal, Safya E Esmaeel, Ritage A Alrawili, Fai B Alanazi, Eman Alanazi, Amani F Alhazimi

**Affiliations:** 1 Department of Pathology, Faculty of Medicine, Northern Border University, Arar, SAU; 2 Department of Physiology, Northern Border University, Arar, SAU; 3 Department of Medicine, Northern Border University, Arar, SAU

**Keywords:** food advertisements, health policy, fast food, childhood obesity, beverage marketing

## Abstract

Background and objective: Children are more susceptible to food and beverage marketing than adults, but little is known about the specific effects of marketing through the media most used by children. This study aims to discover variables that can help inform childhood obesity prevention strategies. Our findings indicate an association between food advertisements and children’s consumption, evidencing a need for the concerned authorities to create strict guidelines that consider the nutritional value of advertised foods.

This study aims to study the attitudes and practices of children related to their preference for unhealthy meals due to food marketing and their association with childhood obesity.

Methodology: A cross-sectional study of randomly selected guardians of children who were screened for obesity. A structured questionnaire was given to the children’s parents.

Results: The study found that most of the participants’ children prefer fast food (291, 78.0%), eat healthy meals (287, 76.9%), and eat fruits and vegetables every day (198, 53.1%). Furthermore, most participants (340, 91.2%) indicated that they were aware of unhealthy diets, and 105 (28.2%) said their children were overweight. Most participants (326, 87.4%) also indicated that watching television (TV) was associated with eating high-calorie foods.

Conclusions: There is strong evidence that children exposed to food marketing develop attitudes about and choose unlimited healthy food and unhealthy foods, which negatively impacts their health. It is recommended that future research employs a wide range of methodologies to study contemporaneous marketing strategies.

## Introduction

According to the latest report from the World Health Organization, the prevalence of obesity among children has increased [1,2]. One of the causes of this increase is the relationship between advertising and high-calorie food intake [3-6]. Communication media, including television (TV), is the primary means of marketing food and beverages to children, as it is highly effective [7]. Some advertisements for food with little nutritional value mislead even consumers who believe in healthy products [8], while others from companies in the food and beverage industry fail to adhere to regulations [9]. It has been highly recommended that advertisements of products low in nutritional value should be restricted [10,11], as childhood obesity can be reduced if the advertising of unhealthy food is limited [12]. However, little is known about the specific effects of marketing through the types of media mostly used by children [13]. Advertising for low-nutrition food uses more visual and audio effects than advertisements for typically nutritious food [14,15], and viewing food advertisements activates areas in the brain related to decision-making. In addition, fictional characters popular among children are combined with messages about health and nutritional quality in food advertisements [16]. Because children respond to these attention-grabbing advertisements, there are negative ramifications for their health, including obesity. There is also concern for people who seek to supplement their emotional needs through food intake (i.e., *emotional eaters*), especially with energy-dense foods with low nutritional value [17]. Children’s responsiveness to the marketing of unhealthy foods is an unsolved problem, and so we aim to study the attitudes and practices of children related to their preferences for unhealthy meals due to food advertising and their association with childhood obesity, specifically in Arar, Kingdom of Saudi Arabia (KSA).

## Materials and methods

Study design and participants

A cross-sectional study of randomly selected guardians (i.e., mothers, fathers, and grandparents) of children who were screened for obesity through an online survey. Children aged below 12 living in the Northern Border Region were included in our study, and children aged over 12 and those who had an infectious disease like bronchitis were excluded. Informed consent was obtained from all the participants, and ethical clearance was obtained from the ethical committee of the College of Medicine at Northern Border University before the commencement of the study.

The study was conducted from October 2023 until April 2024 and assessed the attitudes and practices of children related to their preferences for unhealthy meals due to food advertising. A structured questionnaire was given to the children’s parents.

The survey questions were adopted from the previous studies [2, 7] and were divided into four sections:

Demographic details of the child, including age, sex, weight, and height

Awareness of the parents about unhealthy meals by asking about daily children’s eat and preferred fast food

Associated risk factors for obesity include (eating unhealthy food, less vegetables and fruit, and less physical activity ), its complications, and the child’s history of diabetes

Perceptions and attitudes toward watching food advertisements and buying meals by asking parents about their knowledge of the concept used in advertisements and asking them about the relationship between television watching and high-calorie food intake.

Anthropometric data, including body mass index (BMI), was statistically calculated.

The categories of BMI [2] included the following ranges:

· Underweight: 16.0 to 18.5 (53.5-59.9 kg)

· Normal weight: 18.5 to 25 (60-80.9 kg)

· Overweight: 25 to 30 (81-96.9 kg)

· Obese Class I: 30 to 35 (97-112.9 kg)

· Obese Class II: 35 to 40 (113-129.9 kg)

· Obese Class III: over 40 (over 130 kg)

Statistics

Data were obtained for analysis using IBM SPSS Statistics for Windows, Version 20.0 (IBM Corp., Armonk, NY). The results were presented as percentages and frequency distribution. The chi-square test was used to assess the association between various categorical variables. A *P*-value smaller than 0.05 was considered significant.

## Results

Table 1 shows that of the 373 children who participated in the study through their guardians, 57 (15.3%) were aged between two and four years and the majority (138, 37.0%) were aged between 10 and 12. There were 204 (54.7%) females and 169 (45.3%) males. Of the participants, the majority (338, 90.6%) were Saudi nationals. The distribution of height was as follows: 71 (19.0%) were 51-75 cm, and 84 (22.5%) were 126-150 cm. The participants’ mean weight was 35.02 ± 18.56 kg. Just over half of the children (206, 55.3%) attended school. Of the parents, 79 (21.2%) had completed their secondary education and 265 (71.0%) had their advanced degree. Regarding employment, 91 (24.4%) of the parents were working, 107 (28.7%) were teachers, and 50 (13.4%) held other occupations such as businessperson, doctor, nurse, pharmacist, and technicians or were students.

**Table 1:** Demographic characteristics of the participants’ children (n = 373). Data are presented as *n*, %, and mean ± standard deviation (SD).

Characteristics	Category	*n* (%)
Age (years)	2-4 years	57 (15.3%)
	4-6 years	48 (12.9%)
	6-8 years	65 (17.4%)
	8-10 years	65 (17.4%)
	10-12 years	138 (37.0%)
Sex	Male	169 (45.3%)
	Female	204 (54.7%)
Nationality	Saudi	338 (90.6%)
	Non-Saudi	35 (9.4%)
Height	<50	1 (0.3%)
	50-75 cm	71 (19.0%)
	76-100 cm	69 (18.5%)
	101-125 cm	81 (21.7%)
	126-150 cm	84 (22.5%)
	>150 cm	49 (13.1%)
Weight (kg)	Mean ± SD	35.02 ± 18.56
Does the child attend school?	Yes	206 (55.3%)
	No	169 (44.7%)
Education of parents	Primary	15 (4.0%)
	Intermediate	14 (3.8%)
	Secondary	79 (21.2%)
	Higher degree	265 (71.0%)
Parent job	Employed	91 (24.4%)
	Teacher	107 (28.7%)
	Housewife	125 (33.5%)
	Other specify (business persons, students, doctors, nurses, pharmacists, and technicians)	50 (13.4%)

Table 2 shows that according to most participants in the survey, their child preferred fast food (291, 78.0%), ate healthy meals (287, 76.9%), and ate fruits and vegetables every day (198, 53.1%). A vast majority of the participants (340, 91.2%) indicated that they were aware of unhealthy diets, and only 105 (28.2%) said their child was overweight. Regarding physical activity, 168 participants (77.2%) said their child is active most of the time, while 48.3% said their child participates in physical activity once a week. Of those who exercise, 45 (25.0%) do so every day, 77 (42.8%) do so once a week, and 40 (22.2%) do so occasionally. In addition, 281 (75.3%) participants reported that their child engages in only moderate physical activity for the majority of the week, compared to 78 (27.8%) doing so daily, 126 (44.8%) doing so weekly, 42 (14.9%) doing so occasionally, and 35 (12.5%) doing so at other times. Finally, 106 (28.4%) participants mentioned that their children engaged in intense physical exercise once or twice a week.

**Table 2:** Awareness of unhealthy meals, associated risk factors for obesity, and their complications. Data are presented as *n* and %.

Characteristics	Category	Yes, *n* (%)	No, *n* (%)	I don’t know, *n* (%)
Awareness of unhealthy meals and associated risk factors for obesity and its complications	Does the child eat healthy meals?	287 (76.9%)	71 (19.1%)	15 (4.0%)
	Does the child prefer fast food?	291 (78.0%)	63 (16.9%)	19 (5.1%)
	Does the child eat fruits and vegetables daily?	198 (53.1%)	163 (43.7%)	12 (3.2%)
	Do you know about unhealthy diets?	340 (91.2%)	23 (6.3%)	10 (2.7%)
	Is the child overweight?	105 (28.2%)	255 (68.3%)	13 (3.5%)
	Does the child try to lose weight?	168 (45.0%)	191 (51.2%)	14 (3.8%)
Activity level	Is the child active in general?	288 (77.2%)	78 (20.8%)	7 (1.9%)
	Does the child have weekly physical activity?	180 (48.3%)	182 (48.8%)	11 (2.9%)
	If yes, how many times?			
	Daily	45 (25.0%)	0 (0.0%)	0 (0.0%)
	Weekly	77 (42.8%)	0 (0.0%)	0 (0.0%)
	Once in a while	40 (22.2%)	0 (0.0%)	0 (0.0%)
	Other	18 (10.0%)	0 (0.0%)	0 (0.0%)
	Does the child have only light physical activity in most weeks?	281 (75.3%)	80 (21.4%)	12 (3.2%)
	If yes, how many times?			
	Daily	78 (27.8%)	0 (0.0%)	0 (0.0%)
	Weekly	126 (44.8%)	0 (0.0%)	0 (0.0%)
	Once in a while	42 (14.9%)	0 (0.0%)	0 (0.0%)
	Other	35 (12.5%)	0 (0.0%)	0 (0.0%)
	Does the child have vigorous physical activity once or twice a week?	106 (28.4%)	252 (67.6%)	15 (4.0%)

Table 3 shows that 53 (14.2%) children of the participants had a chronic illness. About half of the participants (187, 50.1%) had a family history of diabetes mellitus. Only 27 (7.2%) parents reported that their child was currently diagnosed with diabetes, which is a lower percentage than the other parents. Of the children with diabetes, most (21, 77.7%) had only received a diagnosis within the last 12 months, and 28 (7.5%) were receiving treatment, either using insulin (15, 53.6%) or another drug (13, 46.4%). Furthermore, 13 (19.6%) of the young diabetics were adhering to dietary guidelines. Regarding obesity prevention, 329 (88.2%) participants knew that eating fewer unhealthy meal patterns could lower the risk of obesity, while 257 (68.9%) were aware that eating a lot of red meat raises the risk of obesity. A significant number of the participants (307, 82.3%) knew that commercially processed foods often have low nutritional value and that eating dietary fiber (320, 85.8%) and fruits and vegetables (340, 91.2%) can reduce the risk of obesity. The higher risk of heart attack, stroke, and cardiovascular disease linked to obesity was also known to the participants; 327 (87.7%) and 352 (94.4%), respectively, acknowledged this risk.

**Table 3:** History of Diabetes Among the Participants’ Children. Data has been presented as n and %

Characteristics	Category	Yes n (%)	No n (%)	I Don’t Know n (%)
History of diabetes	Does the child have chronic Illness?	53 (14.2%)	318 (85.3%)	2 (0.5%)
	Is there a family history of diabetes mellitus?	187 (50.1%)	178 (47.7%)	8 (2.2%)
	Does the child have diabetes mellitus?	27 (7.2%)	339 (90.9%)	7 (1.9%)
	If yes, for how long?			
	Less than 10 years	21 (77.7%)	0 (0.0%)	0 (0.0%)
	More than 10 years	6 (22.3%)	0 (0.0%)	0 (0.0%)
	If yes, does the child take diabetes treatment?	28 (7.5%)	340 (91.2%)	5 (1.3%)
	If yes, which treatment does the child take?			
	Insulin	15 (53.6%)	0 (0.0%)	0 (0.0%)
	Other medication	13 (46.4%)	0 (0.0%)	0 (0.0%)
	Does the child follow diet restrictions due to diabetes mellitus?	73 (19.6%)	289 (77.5%)	11 (2.9%)
	Do you know that you can reduce your risk of obesity by avoiding unhealthy meal patterns?	329 (88.2%)	40 (10.7%)	4 (1.1%)
	Do you know that eating a lot of red meat increases child obesity risk?	257 (68.9%)	97 (26.0%)	19 (5.1%)
	Do you know that foods classified as processed, with low nutritional value due to their high sugar, fat, or salt content, dominate advertisements?	307 (82.3%)	48 (12.9%)	18 (4.8%)
	Do you know that dietary fiber lowers the risk of obesity?	320 (85.8%)	37 (9.9%)	16 (4.3%)
	Do you know that fruits and vegetables prevent the risk of obesity?	340 (91.2%)	28(7.5%)	5(1.3%)
	Do you know that people who are obese are at higher risk of having a heart attack or stroke?	327(87.7%)	32(8.6%)	14(3.7%)
	Do you know that the obese are more susceptible to cardiovascular disease if they are overweight or obese?	352(94.4%)	16(4.3%)	5(1.3%)

Table 4 shows that the majority of the participants (299, 80.2%) agreed that terms like success, enjoyment, adventure, and fun appear in advertisements for low-nutrition products. Furthermore, over half of the participants (219, 58.7%) considered food items with inadequate or nonexistent nutritional value ineffective in averting obesity. Participants (216, 57.9%) also believed that food advertisements conveyed the idea that their children would be popular with their peers by purchasing or consuming food items. Many participants (306, 82.0%) agreed that well-known characters in advertisements targeting children under the age of 12 influenced their decision to buy the product. A majority of the participants (326, 87.4%) thought that watching TV was associated with eating high-calorie foods, and 265 (71.0%) believed that advertisers primarily use TV to market food and beverages, particularly to children. Participants also agreed that, to lower the rates of childhood obesity, TV advertisements for low-nutrition goods should be limited (318, 85.3%) and that their decision-making when making a purchase was strongly influenced by advertisements’ use of pleasure and fulfilling experiences in their messaging (285, 76.4%). A majority of the participants (319, 85.5%) believed that the government should require food and beverage advertisers to alert consumers to the potential health risks associated with particular food or beverage types or classes. In addition, most of the participants (284, 76.1%) thought that *emotional eaters* eat high-energy foods when they experience negative emotions to a greater extent than the general population.

**Table 4:** Perceptions and attitudes toward watching food advertisements and buying meals. Data are presented as *n* and %.

Characteristics	Category	True, *n* (%)	False, *n* (%)	I don’t know, *n* (%)
Perceptions and attitudes regarding watching food advertisements and buying meals	Do you know that success, happiness, adventure, and fun are some of the concepts used in the advertising of products with low nutritional value?	299 (80.2%)	43 (11.5%)	31 (8.3%)
	Do you correspond to food products with low or no nutritional quality, which never helps to prevent obesity?	219 (58.7%)	107 (28.7%)	47 (12.6%)
	Do food advertisements give the impression that consuming food products will make your child popular among his/her friends?	216 (57.9%)	126 (33.8%)	31 (8.3%)
	Do you think that, in advertising aimed at children under 12 years, popular characters influence the marketing of the product?	306 (82.0%)	49 (13.2%)	18 (4.8%)
	Do you think about the relationship between television (TV) watching and high-calorie food intake?	326 (87.4%)	30 (8.0%)	17 (4.6%)
	Do you think that TV is the channel most widely used by advertisers to market food and beverages, since it is highly effective among children, especially at an early age?	265 (71.0%)	87 (23.4%)	21 (5.6%)
	Do you agree that TV advertising of products of low nutritional quality should be restricted to reduce childhood obesity rates?	318 (85.3%)	38 (10.1%)	17 (4.6%)
	Do you think that the purchasing decision process is strongly associated with the message of the ad, which is linked with pleasure and rewarding experiences?	285 (76.4%)	51 (13.7%)	37 (9.9%)
	Do you think that “emotional eaters” consume energy-dense foods with low nutritional value in response to negative emotions to a greater extent than the general population?	284 (76.1%)	39 (10.5%)	50 (13.4%)
	Do you think that the government has a responsible to alert the public to the health problems related to a specific type or class of food or beverage advertisement?	319 (85.5%)	27 (7.2%)	27 (7.2%)

Table 5 shows a statistically significant association between the age of the child and their BMI categories (*P* = 0.005). Among all age groups, children aged between 10 and 12 were the most likely to be overweight or obese. Gender was also a significant factor (*P* = 0.013), with a higher proportion of females classified as overweight or obese. Nationality and BMI categories were also significantly associated (*P* = 0.002), with a greater percentage of non-Saudi children classified as more obese than Saudi children. A child’s BMI category was also significantly associated with their parents’ education levels (*P *= 0.021): the higher the parents’ level of educational attainment, the higher the percentage of children classified in the overweight or obese categories.

**Table 5:** Association between demographic information and body mass index (BMI) of the children (n = 373). Data are presented as *n* and %; a *P*-value < 0.05 was considered statistically significant.

Category	Underweight, *n* (%)	Normal, *n* (%)	Overweight, *n* (%)	Obese I, *n* (%)	Obese II, *n* (%)	Obese III, *n* (%)	*P*-value*
Age (years)							0.005
2-4 years	0 (0.0%)	57 (100%)	0 (0.0%)	0 (0.0%)	0 (0.0%)	0 (0.0%)	
4-6 years	0 (0.0%)	48 (100%)	0 (0.0%)	0 (0.0%)	0 (0.0%)	0 (0.0%)	
6-8 years	0 (0.0%)	65 (100%)	0 (0.0%)	0 (0.0%)	0 (0.0%)	0 (0.0%)	
8-10 years	2 (3.1%)	63 (96.9%)	0 (0.0%)	0 (0.0%)	0 (0.0%)	0 (0.0%)	
10-12 years	18 (13.0%)	113 (81.9%)	3 (2.2%)	1 (0.7%)	0 (0.0%)	1 (0.7%)	
Sex, *n* (%)							0.013
Male	1 (0.6%)	166 (98.2%)	0 (0.0%)	1 (0.6%)	0 (0.0%)	1 (0.6%)	
Female	19 (9.3%)	179 (87.7%)	3 (1.5%)	1 (0.5%)	1 (0.5%)	1 (0.5%)	
Nationality, *n* (%)							0.002
Saudi	18 (5.2%)	314 (92.8%)	3 (0.8%)	1 (0.2%)	1 (0.2%)	1 (0.2%)	
Non-Saudi	2 (5.7%)	32 (91.4%)	0 (0.0%)	0 (0.0%)	0 (0.0%)	1 (2.9%)	
Parent education, *n* (%)							0.021
Primary	1 (6.7%)	13 (86.7%)	0 (0.0%)	1 (6.7%)	0 (0.0%)	0 (0.0%)	
Intermediate	0 (0.0%)	14 (100%)	0 (0.0%)	0 (0.0%)	0 (0.0%)	0 (0.0%)	
Secondary	6 (7.6%)	73 (92.4%)	0 (0.0%)	0 (0.0%)	0 (0.0%)	0 (0.0%)	
Higher degree	13 (4.9%)	246 (92.8%)	0 (0.0%)	1 (0.4%)	0 (0.0%)	2 (0.8%)	
Parent occupation, *n* (%)							0.793
Employed	6 (6.6%)	83 (84.4%)	1 (1.1%)	0 (0.0%)	0 (0.0%)	1 (1.1%)	
Teacher	7 (6.7%)	97 (90.7%)	1 (0.9%)	1 (0.9%)	1 (0.9%)	0 (0.0%)	
Housewife	4 (3.2%)	120 (96.0%)	0 (0.0%)	0 (0.0%)	0 (0.0%)	1 (0.8%)	
Other specify (business persons, students, doctors, nurses, pharmacists, and technicians)	3 (6.0%)	46 (92%)	1 (2.0%)	0 (0.0%)	0 (0.0%)	0 (0.0%)	

## Discussion

We aimed to evaluate the perception and impact of food and beverage marketing on children’s eating behaviors and associated health issues. The findings of the study indicate that most children prefer fast food, eat healthy meals, and eat fruits and vegetables every day and that most children are not overweight. Furthermore, most parents are aware of unhealthy diets. Notably, the study found a gender disparity in regard to BMI, with a higher proportion of females being classified as overweight or obese. Similar to these findings, the study by Castonguay et al. indicated the existence of gender-based disparities in how food marketing affects people’s food intake, choices, and preferences, reactions to particular marketing tactics, attitudes and perceptions about food marketing, the need for regulation, and exposure to and the content of advertisements [18]. A study by Smith et al. also found that a wide range of marketing strategies, especially those employed in television, movies, and product packaging, lead to significant negative effects, including improved attitudes toward, preferences for, and increased consumption of marketed foods, especially among minors [19], indicating a greater tendency among children to consume advertised food compared to adults.

As found in this study, parents are generally aware that commercially processed foods often have low nutritional value. Parents also understand that to reduce the risk of obesity, eating dietary fiber and fruits and vegetables is essential. Despite this, as noted by Driessen et al., parents are not confident in their ability to feed their children healthy foods as a result of the variety of food products advertised more often through social media platforms. In addition, parents frequently underestimate their children’s exposure to and the effects of unhealthy food marketing, particularly in the digital environment [20]. A lack of oversight by parents means that more children are being exposed to unhealthy foods online.

This study also found a statistically significant relationship between children’s age and their BMI category, with children aged between 10 and 12 being more likely to be overweight or obese compared to younger age groups. This finding was corroborated by a study by Bragg et al., which noted that adolescent Black children were more attracted to food and beverage advertisements featuring White people as opposed to Black people and vice versa. On the other hand, the younger group of children was found to be attracted by the food products consumed by their peers [21], implying that the older children were, the more predisposed they were to consuming unhealthy foods.

In addition, the study found that watching TV is linked with eating high-calorie foods, since TV is the primary channel through which advertisers market food and beverages, particularly to children. It was also found that parents widely agree that, to lower the rates of childhood obesity, TV advertisements for low-nutrition goods should be limited, considering that their decision-making when making a purchase was strongly influenced by advertisements’ use of pleasure and fulfilling experiences in their messaging. These findings are similar to a study by Srivastava and Gupta, which found that the widespread promotion of food and beverages aimed at children is a significant contributor to their unhealthy eating habits. Stakeholders are concerned about the strategy employed by companies to actively market unhealthy foods to minors [22]. One aspect of this strategy is celebrity endorsements. According to Zhou et al., celebrity endorsements should be exclusively utilized to promote healthy eating to lower the risk of obesity among young Americans [23].

Nationality was also found to be a significant factor, with a greater percentage of Saudi children classified as more obese compared to non-Saudi children.

This study also examined *emotional eaters*, those who eat energy-dense foods when they experience negative emotions to a greater extent than the general population. Boyland et al. found that females eat more high-energy foods when they are depressed compared to males, who consume more alcohol when they are emotionally distracted or stressed [24]. These findings indicate that the inability to control one’s emotions can contribute to the consumption of unhealthy foods and beverages advertised to the younger generations.

The study also found a relationship between children’s BMI categories and their parents’ education levels. Children of higher educated parents are more likely to be overweight or obese. As found by Chiong and Figueroa, parental favorability toward food and beverage advertisements influences unhealthy food consumption among children. However, this association changes when food insecurity is taken into consideration. Chiong and Figueroa also found that parents with a higher level of education are more likely to explore advertised food products, which increases their children’s consumption of these products [25]. This implies that several risk factors associated with advertised food and beverage consumption contribute to health issues among children.

The current research has several limitations. First, it did not consider parents’ susceptibility to different marketing strategies aimed at children. Parents may react differently depending on their ages, gender, cultural background, and socioeconomic status. Another limitation is that it did not consider how other factors, such as parental attitudes and the availability of healthful food options, affect children’s eating behavior. These external variables may have a significant impact on how children chose to eat and on their overall health. These limitations should be taken into account when analyzing the findings and their implications for public health interventions.

## Conclusions

This study has examined the role of food marketing in children developing attitudes about and making choices about unlimited healthy and unhealthy foods and the associated negative health effects. The study found strong evidence to support the restriction of food marketing to children. It is recommended that future research employ a wide range of methodologies to study contemporaneous marketing strategies.

## References

[1] AlEnazi S, AlAjlan R, AlKhalaf H (2023). Prevalence of obesity among children and adolescents in Saudi Arabia: a multicenter population-based study. Saudi J Med Med Sci.

[2] Alsulami S, Baig M, Ahmad T (2023). Obesity prevalence, physical activity, and dietary practices among adults in Saudi Arabia. Front Public Health.

[3] Davó-Blanes MC, Ortiz-Moncada R, Gil-González D, Alvarez-Dardet C, Lobstein T (2013). The impact of marketing practices and its regulation policies on childhood obesity. Opinions of stakeholders in Spain. Appetite.

[4] Montaña M, Jiménez-Morales M, Vàzquez M (2019). Food advertising and prevention of childhood obesity in Spain: Analysis of the nutritional value of the products and discursive strategies used in the ads most viewed by children. Nutrients.

[5] Lake AA (2018). Neighbourhood food environments: food choice, foodscapes and planning for health. Proc Nutr Soc.

[6] Sonntag D, Schneider S, Mdege N, Ali S, Schmidt B (2015). Beyond food promotion: a systematic review on the influence of the food industry on obesity-related dietary behaviour among children. Nutrients.

[7] Pauzé E, Potvin Kent M (2021). Children's measured exposure to food and beverage advertising on television in Toronto (Canada), May 2011-May 2019. Can J Public Health.

[8] Iye R, Okuhara T, Okada H, Yokota R, Kiuchi T (2021). A content analysis of video advertisements for dietary supplements in Japan. Healthcare (Basel).

[9] Watson WL, Johnston A, Hughes C, Chapman K (2014). Determining the "healthiness" of foods marketed to children on television using the Food Standards Australia New Zealand nutrient profiling criteria. Nutr Diet.

[10] Arenaza L, Muñoz-Hernández V, Medrano M (2018). Association of breakfast quality and energy density with cardiometabolic risk factors in overweight/obese children: role of physical activity. Nutrients.

[11] León-Flández K, Rico-Gómez A, Moya-Geromin MÁ (2017). Evaluation of compliance with the Spanish Code of self-regulation of food and drinks advertising directed at children under the age of 12 years in Spain, 2012. Public Health.

[12] Veerman JL, Van Beeck EF, Barendregt JJ, Mackenbach JP (2009). By how much would limiting TV food advertising reduce childhood obesity?. Eur J Public Health.

[13] Théodore FL, López-Santiago M, Cruz-Casarrubias C, Mendoza-Pablo PA, Barquera S, Tolentino-Mayo L (2021). Digital marketing of products with poor nutritional quality: a major threat for children and adolescents. Public Health.

[14] Bozzola E, Spina G, Agostiniani R (2022). The use of social media in children and adolescents: Scoping review on the potential risks. Int J Environ Res Public Health.

[15] Alsharif AH, Salleh NZ, Al-Zahrani SA, Khraiwish A (2022). Consumer behaviour to be considered in advertising: A systematic analysis and future agenda. Behav Sci (Basel).

[16] Hyuksoo K, Doohwang L, Yangsun H, Jungsun A, Ki-Young L (2016). A content analysis of television food advertising to children: comparing low and general-nutrition food. Int J Consum Stud.

[17] Silva P, Araújo R, Lopes F, Ray S (2023). Nutrition and food literacy: framing the challenges to health communication. Nutrients.

[18] Castonguay J, Kunkel D, Wright P, Duff C (2013). Healthy characters? An investigation of marketing practices in children's food advertising. J Nutr Educ Behav.

[19] Smith R, Kelly B, Yeatman H, Boyland E (2019). Food marketing influences children’s attitudes, preferences and consumption: a systematic critical review. Nutrients.

[20] Driessen C, Kelly B, Sing F, Backholer K (2022). Parents’ perceptions of children’s exposure to unhealthy food marketing: a narrative review of the literature. Curr Nutr Rep.

[21] Bragg MA, Miller AN, Kalkstein DA, Elbel B, Roberto CA (2019). Evaluating the influence of racially targeted food and beverage advertisements on Black and White adolescents' perceptions and preferences. Appetite.

[22] Srivastava Srivastava, R. R., Gupta Gupta, P P (2022). Research on unhealthy food and beverages advertising targeting children: systematic literature review and directions for future research. Aust J Manage.

[23] Zhou M, Rincón-Gallardo Patiño S, Hedrick VE, Kraak VI (2020). An accountability evaluation for the responsible use of celebrity endorsement by the food and beverage industry to promote healthy food environments for young Americans: a narrative review to inform obesity prevention policy. Obes Rev.

[24] Boyland E, McGale L, Maden M, Hounsome J, Boland A, Angus K, Jones A (2022). Association of food and nonalcoholic beverage marketing with children and adolescents’ eating behaviors and health: a systematic review and meta-analysis. JAMA Pediatr.

[25] Chiong R, Figueroa R (2022). Food insecurity and the association between perceptions and trust of food advertisements and consumption of ultra-processed foods among U.S. parents and adolescents. Nutrients.

